# Attachment to Home and Community in Older Rural African Americans in Mississippi

**DOI:** 10.1093/geroni/igaa057.1052

**Published:** 2020-12-16

**Authors:** Carolyn Adams-Price, Muhammed Riaz, Margaret Ralston, Antonio Gardner

**Affiliations:** Mississippi State University, Mississippi State, Mississippi, United States

## Abstract

The purpose of this study was to examine qualitatively attachment to home and community in older rural African Americans in the Deep South. Sixty adults aged 52-79 (mean age 64.7, 24 males and 36 females) were interviewed using semi-structured interviews. Participants lived in two micropolitan counties in Mississippi, with most living in one of two mostly African American communities with fewer than 1000 residents. Interviewees were asked about their attachment to their house, the land the house is on, and the community in which they live. Interviews were recorded and transcribed, and transcriptions were analyzed for themes by two qualitative researchers using phenomenological analysis. The two researchers uncovered similar themes; discrepancies were discussed and integrated, and checked for consistency with the original text. The most prominent themes for attachment to home were sense of ownership/having built or made home their own, legacy/generational/historical attachment, sense of peace and safety, solitude and privacy, and attachment to specific features. Interviewees felt that their homes were a part of who they are and a part of their personal and family history; they also reported that their homes were safe and comfortable havens. When participants were asked about their attachment to their community, four themes emerged: socialization/friendly visiting, family and close ties, religious/spiritual, and solitude/quiet community. These results will be discussed in the context of Wahl’s 2012 model, which asserts that older adults’ attachment to place is a function of agency and belonging; belonging was a more prominent theme in this group.

